# Shaping the Bioactive Properties of Kombucha Drinks by Using Raw Materials Alternative to Tea

**DOI:** 10.3390/molecules31071170

**Published:** 2026-04-01

**Authors:** Akshay Chandran, Joanna Wyka, Gloria-Renate Klein, Barbara Stefanska, Joanna Kolniak-Ostek

**Affiliations:** 1 Department of Fruit, Vegetable and Plant Nutraceutical Technology, Wrocław University of Environmental and Life Sciences, 51-630 Wrocław, Poland; 2 Department of Human Nutrition, Faculty of Biotechnology and Food Science, Wrocław University of Environmental and Life Sciences, 51-630 Wrocław, Poland; joanna.wyka@upwr.edu.pl; 3 Food, Nutrition and Health Program, Faculty of Land and Food Systems, The University of British Columbia, Vancouver, BC V6T 1Z4, Canada; gloklei@student.ubc.ca (G.-R.K.); barbara.stefanska@ubc.ca (B.S.)

**Keywords:** kombucha, alternative substrates, bioactive compounds, antioxidant activity, metabolic health, waste valorization

## Abstract

Alternative substrates to traditional *Camellia sinensis* tea are increasingly investigated to diversify kombucha and enhance its functional properties. This review synthesizes evidence (2020–2025) on how non-tea substrates influence microbial ecology, metabolite composition, and bioactivity of kombucha. A semi-systematic search of PubMed, Scopus, Web of Science, and publisher platforms identified studies on fruit, vegetable, herbal, algal, cereal, dairy, and food-industry by-product substrates reporting compositional or functional outcomes. Extracted data included substrate characteristics, fermentation conditions, SCOBY features, analytical methods, and reported antioxidant, anti-inflammatory, metabolic, probiotic, and dermatological effects. Fermentation often leads to an increase in total phenolic content and antioxidant capacity. These effects are highly dependent on fermentation conditions, particularly duration and substrate composition. In some cases, prolonged fermentation may result in phenolic degradation or transformation, leading to reduced levels of certain compounds. Fruit- and hibiscus-based systems enhanced anthocyanin-driven antioxidant and anti-inflammatory activity. Vegetable and cereal substrates supplied phenolic acids and *β*-glucans associated with metabolic regulation and gut health, whereas by-products and algal fermentations supported waste valorization and enrichment in chlorogenic acids, pigments, fibers, and peptides. Despite promising functionality, substantial inter-study variability and limited in vivo validation and the lack of standardized fermentation protocols constrain translational application. In addition, the inherent variability in SCOBY microbial composition represents a major source of inconsistency, as differences in microbial communities can significantly influence fermentation dynamics, metabolite profiles, and functional outcomes.

## 1. Introduction

Fermented functional beverages continue to gain global attention due to their association with gut health, metabolic regulation, and natural bioactive compounds [1,2]. Among these, kombucha, a fermented beverage traditionally produced from sweetened *Camellia sinensis* infusions and a symbiotic culture of bacteria and yeasts (SCOBY), has become widely recognized for its antioxidant and antimicrobial properties, as well as its potential probiotic-like effects. In contrast, most microorganisms present in SCOBY, particularly acetic acid bacteria, are not recognized as established probiotics, and their functional relevance in humans remains to be confirmed [3]. During fermentation, microbial metabolism transforms sugars and tea polyphenols into organic acids, phenolic derivatives, and other metabolites that contribute to the functional profile of the fermented beverage [2,3].

Growing commercial interest has accelerated research on the biochemical and technological characteristics of kombucha. The global kombucha market was valued at USD 4.26 billion in 2024 and is projected to exceed USD 9 billion by 2030 [4], reflecting the increasing consumer demand for minimally processed, health-oriented beverages. Despite this rapid growth, tea-based kombucha supports a rich and complex network of polyphenol-derived metabolites, as black or green tea primarily contributes catechins, theaflavins, and simple phenolic acids [1]. Nevertheless, the range and variability of bioactive compounds can be further expanded through the use of alternative substrates. This has stimulated innovation in substrate selection, moving beyond traditional tea toward matrices that may enhance nutritional value, diversify sensory properties, or support sustainable production practices [5]. Alternative substrates, including fruits, fruit juices, vegetables, herbs, algae, cereals, and food industry by-products, have emerged as promising foundations for kombucha fermentation.

At the microbial and biochemical level, substrate composition influences both microbial succession and metabolic fluxes within the SCOBY consortium. Yeasts, including *Saccharomyces* and *Zygosaccharomyces*, initiate sucrose hydrolysis via invertase activity and metabolize the resulting monosaccharides to ethanol, which is subsequently oxidized into organic acids by acetic acid bacteria (*Komagataeibacter* and *Gluconacetobacter*), with Komagataeibacter often representing the dominant genus within the bacterial fraction of SCOBY [3]. Phenolic compounds, glycosides, pigments, and other precursors undergo microbial hydrolysis, oxidation, reduction, and esterification, generating metabolites with enhanced antioxidant, anti-inflammatory, or metabolic-regulating potential [6]. Although individual studies have highlighted these substrate-driven transformations, comprehensive syntheses bridging substrate composition, microbial activity, and functional outcomes remain limited. While previous reviews have addressed kombucha produced from alternative substrates, many focus on specific substrate categories, microbiology, or general bioactivities [1,2], with limited integration of diverse non-tea fermentations.

Given the expanding diversity of raw materials and the increasing interest in designing kombucha with targeted functional attributes, a structured synthesis is needed to clarify how specific substrate categories modulate microbial metabolism and contribute to bioactive compound formation. Therefore, this review integrates biochemical, microbiological, and functional evidence to examine how alternative substrates shape the bioactive properties of kombucha. Specifically, it (i) outlines the major substrate categories used in kombucha fermentation, (ii) describes the microbial and enzymatic processes by which substrates influence metabolite formation, and (iii) synthesizes evidence on antioxidant, anti-inflammatory, metabolic, and other functional effects across fermentation systems. By framing kombucha as a modular bioprocessing platform, this review highlights opportunities for functional beverage innovation, sustainable raw material utilization, and targeted health-oriented product development.

## 2. Microbial and Biochemical Mechanisms of Kombucha Fermentation

Kombucha fermentation is driven by a complex and dynamic microbial consortium known as the SCOBY (Symbiotic Culture of Bacteria and Yeasts). This self-organizing system enables the extensive biochemical transformation of sugars, amino acids, and phenolic precursors into a range of metabolites, including organic acids, vitamins, peptides, and polyphenol derivatives [2,7].

### 2.1. Composition and Interactions Within the SCOBY Consortium

The SCOBY architecture is composed of a cellulose pellicle synthesized primarily by *Komagataeibacter* spp., forming a porous scaffold at the air–liquid interface that facilitates oxygen diffusion and supports microbial attachment and metabolite exchange [7,8]. In addition to its structural role, the pellicle contributes to the spatial organization and stability of the microbial consortium and provides a protective biofilm-like environment. Importantly, cellulose production also influences oxygen diffusion gradients across the fermentation system, thereby affecting microbial stratification and fermentation kinetics, particularly the balance between yeast-driven ethanol production and bacterial oxidative metabolism. Acetic acid bacteria (AAB), including *Komagataeibacter*, *Acetobacter*, and *Gluconobacter*, occupy this oxygen-rich layer and perform oxidative metabolism, converting ethanol and sugars into acetic, gluconic, and glucuronic acids [9,10]. These acids contribute substantially to the acidity, antimicrobial effects, and potential detoxification functions of beverages.

In parallel, yeasts such as *Zygosaccharomyces*, *Saccharomyces*, *Brettanomyces*, *Pichia*, and *Candida* initiate sucrose breakdown through invertase activity, producing glucose, fructose, and ethanol, which are key substrates for AAB-driven oxidation. This metabolic cross-feeding forms the foundation of a mutualistic loop that maintains microbial stability. Yeasts typically dominate the deeper, less oxygenated layers of the biofilm, whereas AAB thrives near the surface, resulting in a stratified consortium [8] that allows simultaneous anaerobic alcohol production and aerobic oxidation of organic acids.

**Figure 1:**
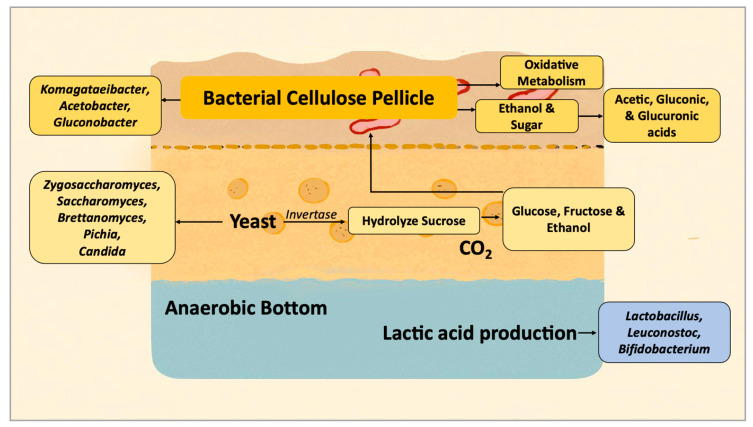
Visual summary of spatial organization and metabolite exchange within the SCOBY biofilm.

Minor bacterial groups, including *Lactobacillus* and *Leuconostoc*, have occasionally been identified in certain kombucha cultures and may contribute to metabolic diversity within the consortium [11]. Reports of *Bifidobacterium* are limited and inconsistent, and its presence in SCOBY remains uncertain. The potential functional roles of these microorganisms require further validation, as their probiotic effects have not been confirmed in vivo [10]. Although their abundance is typically lower than that of AAB, these taxa may interact with substrate components (e.g., fibers and oligosaccharides) in ways that enhance fermentation efficiency or expand the metabolite spectrum. SCOBY functions as a metabolically integrated, spatially structured biofilm whose interactions are shaped by oxygen gradients, substrate availability, and long-term microbial co-adaptation (Figure 1).

### 2.2. Enzymatic Transformations of Primary Substrates

Kombucha fermentation progresses through a coordinated series of enzymatic reactions linking carbohydrate, phenolic, and nitrogen metabolism. The process is primarily initiated by yeast-derived *β*-fructofuranosidase (invertase), which hydrolyzes sucrose into glucose and fructose; however, certain bacterial species may also contribute to this activity. These monosaccharides enter glycolysis and are converted to ethanol and CO_2_ under semi-anaerobic conditions [2]. Ethanol subsequently becomes the major substrate for acetic acid bacteria (AAB), which oxidize ethanol to acetic acid via alcohol dehydrogenase and acetaldehyde dehydrogenase. In parallel, AAB catalyzes the oxidative transformation of glucose: glucose dehydrogenase yields gluconic acid, whereas glucuronate dehydrogenase contributes to glucuronic acid production [8,12]. These transformations reflect a complex network of interconnected metabolic pathways involving multiple enzymes and intermediate compounds, rather than a single linear process. Together, these pathways generate the characteristic organic acid profile that shapes the acidity, antimicrobial potential, and metabolic effects of kombucha.

**Figure 2:**
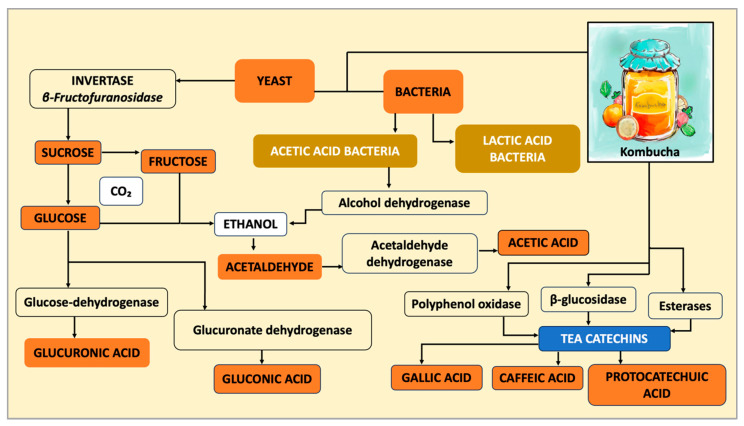
Central biotransformation routes during kombucha fermentation.

In addition to carbohydrate catabolism, microbial enzymes substantially modify phenolic structures. Polyphenol oxidase, *β*-glucosidase, and esterases, produced by both yeasts and bacteria, contribute to the transformation of phenolic compounds. Yeasts are primarily associated with glycoside hydrolysis and the release of bound phenolics, whereas acetic acid bacteria contribute to oxidative transformations, and lactic acid bacteria, when present, may further participate in phenolic modification through reductive and hydrolytic activities [13,14]. These reactions enhance the antioxidant potential and can shift the phenolic fingerprint of the final beverage. The magnitude and direction of phenolic biotransformation are strongly influenced by microbial diversity, enzyme expression, and substrate composition. Matrices enriched in anthocyanins, such as hibiscus, mulberry, or berry-based substrates, exhibit distinct transformation pathways and may yield unique metabolite profiles, including the formation of phenolic acids such as protocatechuic and gallic acids through microbial cleavage and degradation of anthocyanin structures [15]. These biochemical conversions illustrate the central role of microbial enzymes in shaping kombucha’s chemical landscape. Figure 2 summarizes the major biotransformation routes, from initial sucrose cleavage to the formation of organic acids and phenolic derivatives, which contribute to the functional properties of the beverage.

### 2.3. Formation of Secondary Metabolites and Bioactive Compounds

The cooperative metabolism of SCOBY leads to the accumulation of a broad spectrum of secondary metabolites, which determine the functional profile of kombucha. These include organic acids (acetic, gluconic, glucuronic, lactic, and citric acids), which play a role in detoxification and product preservation; phenolic derivatives (gallic, chlorogenic, and caffeic acids), which confer antioxidant and anti-inflammatory properties; amino acids and peptides arising from proteolytic activity or microbial biosynthesis; vitamin B complex which may be synthesized by microbial activity, and vitamin C, whose levels are often influenced by substrate composition and fermentation conditions rather than confirmed de novo synthesis; bacterial cellulose, the primary extracellular polysaccharide produced mainly by *Komagataeibacter*, contributes to the structural and textural properties of kombucha, while other exopolysaccharides, if present, occur in smaller amounts [7,10].

Recent metabolomic profiling has revealed novel fermentation-derived molecules, including various fermentation-derived metabolites, such as modified phenolic compounds and short-chain fatty acids, which may explain the synergistic health effects of kombucha [16,17]. These findings confirm that SCOBY acts as a microbial biotransformation system, converting plant-derived precursors into metabolites with enhanced bioactivity and bioavailability.

### 2.4. Factors Influencing Microbial and Biochemical Dynamics

The balance between yeasts and acetic acid bacteria within SCOBY is highly sensitive to environmental and process-related parameters. Temperature is a primary determinant of microbial dominance: yeast metabolism is generally favored at lower temperatures, although it can remain active above 25 °C depending on species and environmental conditions, whereas AAB activity and cellulose production often peak at approximately 30 °C [1,13]. As fermentation progresses, the pH typically declines to 2.5–3.5, although this range may vary depending on substrate composition and fermentation conditions, with vegetable-based substrates often exhibiting higher pH values due to their buffering capacity. The carbon-to-nitrogen ratio of the substrate further modulates microbial growth, influencing organic acid production, sugar utilization rates, and overall fermentation kinetics. Although prolonged fermentation can increase total phenolic content, it may also lead to excessive acidification and reduced sensory acceptance [8].

Substrate complexity also plays an important role in shaping microbial dynamics. Polyphenol-rich or fiber-dense matrices may influence oxygen diffusion, enzyme accessibility, and microbial attachment, likely through changes in matrix structure, viscosity, and physical properties [3,18]. Substrates abundant in anthocyanins, such as mulberry or hibiscus, may buffer acidification, while polysaccharides from aloe or cereal-based substrates can enhance bacterial cellulose formation and alter the SCOBY structure [19]. These effects reflect substrate-specific interactions, in which physical properties (viscosity and porosity) and biochemical composition (phenolics, fibers, and sugars) collectively shape microbial behavior.

Understanding how substrate characteristics interact with microbial metabolism is essential for guiding kombucha fermentation toward targeted functional outcomes. Ultimately, kombucha represents a self-regulating ecosystem in which metabolic cooperation enables the transformation of sugars, polyphenols, and other precursors into a broad spectrum of bioactive metabolites. The spatial and functional organization of SCOBY ensures continuous cross-feeding between microbial groups, while enzyme-driven reactions define the biochemical fingerprint of the final product [20,21].

## 3. Alternative Substrates for Kombucha Fermentation

A growing body of research demonstrates that fruit juices, plant materials, herbal infusions, algae, mushrooms, and food industry by-products can effectively support SCOBY activity, generating beverages with distinct biochemical signatures and enhanced functional potential [22,23,24].

### 3.1. Fruit and Fruit-Based Substrates

Fruit juices are one of the most extensively studied alternatives for kombucha fermentation due to their high sugar content, favorable acidity, and abundance of phenolic compounds. Their composition supports vigorous yeast metabolism and provides a chemically rich environment for microbial transformation. Fermentations using pomegranate, mulberry, and grape juices consistently show increased total phenolic content, although the magnitude of this increase varies considerably depending on substrate composition, fermentation conditions, and analytical methods [23,25]. These changes have been linked to the microbial cleavage of ellagitannins into gallic and ellagic acids, which have high antioxidant and anti-inflammatory potential.

Similar enhancements have recently been reported for apple and pear substrates, where glucuronidation and microbial hydrolysis contribute to elevated antidiabetic, antioxidant, and anti-inflammatory enzymatic activities [26]. Other fruit-based matrices, including pineapple, mango, as well as plant-based substrates such as jackfruit leaves, offer additional advantages by supplying endogenous enzymes, such as bromelain and amylases, which may contribute to the modification of substrate components and potentially influence fermentation dynamics [27,28]. These systems often yield beverages with balanced acidity and favorable sensory profiles.

Fruit phenolics have also been shown to exert ecological effects on SCOBY. Several studies have indicated that fruit-derived flavonoids and acids promote yeast proliferation while moderating excessive acetic acid formation, improving taste stability and reducing over-acidification [21,29]. Collectively, fruit-based fermentations demonstrate the capacity of alternative substrates to enhance both biochemical functionality and sensory acceptability.

### 3.2. Vegetable and Plant-Based Matrices

Vegetables, roots, and leafy greens provide an alternative set of substrates characterized by phenolic acids, carotenoids, vitamins, and fiber, all of which contribute to nutritional enrichment and functional enhancement during kombucha fermentation. Beetroot-based fermentations may retain significant amounts of betalain pigments; however, their stability is highly dependent on fermentation conditions, and partial degradation is often observed [30]. Similarly, sweet potatoes and spinach leaves, owing to their high chlorogenic acid and flavonoid content, support microbial growth and promote the formation of organic acids, such as malic and citric acids [18].

A notable technological advantage of vegetable matrices is their buffering capacity, which may moderate pH changes during fermentation. Nevertheless, the production of organic acids is primarily driven by microbial metabolism and the availability of fermentable sugars. This effect increases the retention of heat- and pH-sensitive micronutrients, contributing to improved nutritional quality of the food [31]. The fiber and polysaccharides present in many vegetables can further influence fermentation dynamics by functioning as prebiotic substrates. These compounds stimulate bacterial cellulose production by *Komagataeibacter*, improving beverage viscosity and potentially contributing to gastrointestinal benefits [24].

### 3.3. Herbs, Botanicals, and Tea Alternatives

Replacing *Camellia sinensis* with herbal and botanical infusions introduces new functional and sensory attributes to kombucha, including caffeine-free alternatives with distinctive phytochemical profiles. Fermentations based on *Hibiscus sabdariffa*, rooibos, yerba mate, and *Moringa oleifera* frequently exhibit elevated anthocyanin and total phenolic contents. For instance, hibiscus-based kombucha may demonstrate elevated levels of delphinidin and cyanidin derivatives, accompanied by enhanced antioxidant and antimicrobial activities; however, anthocyanin stability is variable, and partial degradation during fermentation is frequently observed depending on processing conditions [32,33,34].

A recent study by Pawluś and Kolniak-Ostek compared mint- and nettle-based kombucha products and reported significant increases in antidiabetic and anti-inflammatory activity, associated with phenolic biotransformation and glucosidase inhibition [35]. Other botanicals, such as rosemary, thyme, and lavender, supply volatile terpenoids with antimicrobial and aromatic properties that diversify sensory profiles while contributing functional benefits [36,37,38]. Additional studies suggest that substrates such as *Clitoria ternatea* and *Rhizophora mucronata* leaves, both rich in anthocyanins and other polyphenols, produce kombucha beverages with promising antidiabetic potential [39,40].

### 3.4. Food Industry By-Products and Waste Valorization

Sustainable approaches in kombucha research increasingly focus on valorizing food industry by-products as unconventional fermentation substrates. Fermentations based on lavender and sage distillation residues have been shown to yield kombucha beverages with strong antioxidant and anti-inflammatory properties, demonstrating that low-value waste streams can support meaningful bioactivities. Nevertheless, such substrates often require formulation adjustments, including sugar supplementation, to provide sufficient fermentable carbon for microbial activity [37]. Likewise, the use of coffee silverskin extracts led to elevated levels of chlorogenic acid and trigonelline derivatives, enhancing the antioxidant profile of the beverage [22]. However, due to its low content of fermentable sugars, coffee silverskin typically requires supplementation (e.g., sucrose addition) to support efficient fermentation. By-products often contain lower intrinsic sugar levels, which can contribute to reduced sugar formulations while still supporting robust fermentation [41].

### 3.5. Algae, Seeds, and Other Emerging Substrates

Recent innovations have expanded kombucha fermentation to unconventional substrates, including microalgae, mushrooms, seeds, and specialized plant-derived matrices. The diversity of alternative substrates and their corresponding bioactive outcomes are summarized in Table 1. Microalgal substrates, such as Spirulina and Chlorella, introduce pigments (phycocyanins and carotenoids), minerals, and bioactive peptides that increase nutritional density and substantially enhance antioxidant capacity [24]. Nevertheless, their high nitrogen content and relatively low levels of fermentable sugars may lead to an imbalanced C:N ratio, posing challenges for efficient SCOBY fermentation. Fermentation with *Ganoderma lucidum* (reishi mushroom) produces beverages enriched with phenolic compounds that exhibit immunomodulatory potential [42].

Seed-based substrates, including hemp, chia, and flax, provide omega-3 fatty acids, which are derived from the raw materials themselves rather than being generated or significantly modified during fermentation, whereas cereal-derived matrices, such as oat, rice, and soy, supply *β*-glucans associated with prebiotic and cholesterol-lowering effects [43,44]. Aloe vera represents another emerging substrate category, offering polysaccharides and antioxidant compounds that contribute to skin-protective and anti-inflammatory properties in the resulting beverages [32].

**Table 1:** Overview of alternative substrates used for kombucha fermentation and their corresponding bioactive and functional outcomes (compiled from the cited literature).

Substrate Type/Example	Key Bioactive Changes After Fermentation	Main Functional Effects	Reference
Fruit-based substrates			
Pomegranate, grape, mulberry,	↑ Total phenolics (30–50%), conversion of ellagitannins to gallic and ellagic acids	↑ antioxidant (DPPH, FRAP, ABTS, T-SOD assay kit), ↑ anti-inflammatory and ↑ cardioprotective activity	[23,26,28]
Apple juices			
Mango and pineapple juices, Jackfruit leaves	Presence of natural hydrolytic enzymes (bromelain, amylases) improving fermentation efficiency	↑ antioxidant (DPPH), balanced acidity, ↑ sensory attributes	[21]
Snake fruit juice	↑ Antioxidants and anti-microbial properties, anti-diabetic properties	↑ antioxidant (DPPH, SOD, MDA), ↑ anti-inflammatory and ↑ antidiabetic properties ↑ antibacterial properties (Agar well diffusion)	[45,46]
Cactus pear	↑ Phenolic compounds and betalains	↑ anti-bacterial activity ↑ antimicrobial activity (Agar well diffusion)	[47]
**Vegetable and plant-based matrices**			
Beetroot	↑ Betalain pigments during fermentation, resulting in beverages rich in betanin and isobetanin.	↑ antioxidant (DPPH) and ↑ anti-inflammatory potential.	[30]
Sweet potato, spinach leaves	↑ Chlorogenic acid and flavonoids, supporting microbial growth and promoting the formation of organic acids such as malic and citric acids	↑ nutritional profile and ↑ antioxidant potential (DPPH, TPC)	[18]
**Food by-products and waste valorization**			
Grape pomace	Retained substantial levels of phenolic compounds and dietary fiber after fermentation.	↑ antioxidant benefits (DPPH) and ↑ digestive health	[37,48,49]
Essential oil distillation waste			
Coffee silverskin	↑ chlorogenic acid and trigonelline derivatives	↑ antioxidant (DPPH, ORAC) and ↑ antidiabetic activity.	[22]
Lavender and sage distillation residues	Exhibit strong antioxidant and anti-inflammatory properties	↑ antioxidant (DPPH) and ↑ anti-inflammatory potential.	[22]
Winery effluent	↑ Antioxidant activity and OH inhibition	Potent free radical scavenging, antioxidative protection (DPPH)	[50]
Pueraria lobata starch production waste water	↑ Antioxidant activity	↑ antioxidant (DPPH), ↑ anti-inflammatory and ↑ cardioprotective activity	[51]
**Algae, fungi, and other emerging substrates**			
Microalgae (Spirulina or Chlorella)	Introduces bioavailable pigments (phytocyanins, carotenoids), minerals, and peptides.	↑ nutritional density and ↑ antioxidant capacity (DPPH)	[24]
*Ganoderma lucidum* (reishi mushroom)	↑ Total phenolics	↑ immunomodulatory potential (DPPH, Folin–Ciocalteu)	[42]
Seed-based kombucha from hemp, chia, or flax	Provide omega-3 fatty acids, fiber, and lignans	↑ heart health, digestion, and ↑ antioxidant effects (DPPH, Folin–Ciocalteu, micro-Lowry’s method).	[43,44]
Cereal-derived matrices (oat, rice, soy whey)	Supply β-glucans.	↑ Prebiotic and ↓ cholesterol effects (micro-Lowry’s method)	[43,44]
Aloe vera	↑ Antioxidant properties.	↑ Skin protective benefits (DPPH, ABTS)	[32]
Mushroom	↑ Anti-inflammatory activity	↓ oxidative stress (PBMC cultures, TPC)	[52]
Sea grapes (*Caulerpa racemosa*)	↑ Lipase inhibitory	Induced weight loss and ↑ levels of liver SOD (Superoxide Dismutase) ↓ Total cholesterol, Triglycerides, ↑ Low Density Lipoprotein, and ↓ High Density Lipoprotein levels. (ELISA, In vitro assay)	[53]

Abbreviations: TPC (Total phenolic content), DPPH (2,2-diphenyl-1-picrylhydrazyl), FRAP (Ferric Reducing Antioxidant Power), ABTS (2,2’-azino-bis (3-ethylbenzothiazoline-6-sulfonic acid)), T-SOD (Total superoxide dismutase), SOD (Superoxide dismutase), MDA (Malondialdehyde), ORAC (Oxygen Radical Absorbance Capacity), PBMC (Peripheral blood mononuclear cells), ELISA (Enzyme-linked immunosorbent assay); ↑—increase; ↓—decrease. Antioxidant assays (e.g., DPPH, ABTS, FRAP) reflect chemical radical-scavenging capacity and should not be directly interpreted as evidence of in vivo biological effects.

### 3.6. Functional and Technological Implications

Alternative substrates fundamentally influence kombucha fermentation by reshaping the microbial activity, enzymatic pathways, and resulting metabolite spectrum. Fruit-based systems tend to amplify antioxidant potential owing to elevated phenolic availability, whereas vegetable matrices enhance pigment stability and vitamin retention. Herbal and floral infusions contribute to distinctive volatile and antimicrobial compounds, and substrates derived from by-products or algae expand the nutritional value of beverages while supporting sustainability-driven innovation [23,24]. From a technological standpoint, substrate composition affects key process parameters such as pH buffering, viscosity, carbonation dynamics, and flavor balance. These factors determine fermentation kinetics, microbial succession, and product stability, enabling the formulation of custom kombucha beverages tailored to specific functional, sensory, or market niches [22,34]. Substrates rich in fibers or polysaccharides, for instance, increase bacterial cellulose production and modulate mouthfeel, whereas phenolic-rich matrices help control excessive acidification and improve sensory acceptability.

Overall, kombucha functions as a flexible bioprocessing platform capable of converting diverse botanical and food-derived resources into multifunctional beverages that integrate health-promoting properties, sustainability, and sensory innovation. The biochemical diversity introduced by alternative substrates ultimately determines the biological activities of the final product.

## 4. Bioactive Potential and Functional Effects

Although traditional tea-based kombucha is widely recognized for its antioxidant and detoxifying properties, the incorporation of alternative plant substrates—fruits, vegetables, herbs, and agricultural by-products—substantially broadens its bioactive profile and can enhance or diversify its functional effects [23,54,55,56]. It should be emphasized that most reported bioactivities are derived from in vitro assays, which may not directly reflect physiological effects in humans. Consequently, these findings should be interpreted with caution, and further in vivo and clinical studies are required to confirm their relevance. The following sections synthesize the current evidence on the principal biological activities associated with kombucha produced from non-tea matrices.

### 4.1. Antioxidant and Radical-Scavenging Activity

Antioxidant capacity is the most extensively characterized functional attribute of kombucha [57,58]. Fermentation enhances radical-scavenging potential through multiple interacting mechanisms, including the release and transformation of phenolic compounds, the formation of new low-molecular-weight metabolites, and changes in compound bioavailability. First, microbial hydrolysis and oxidation release phenolic compounds from complex plant matrices and convert them into more bioavailable derivatives. Second, fermentation generates low-molecular-weight antioxidants, including organic acids, peptides, and vitamin C, which further contribute to the total antioxidant capacity [59,60].

**Figure 3:**
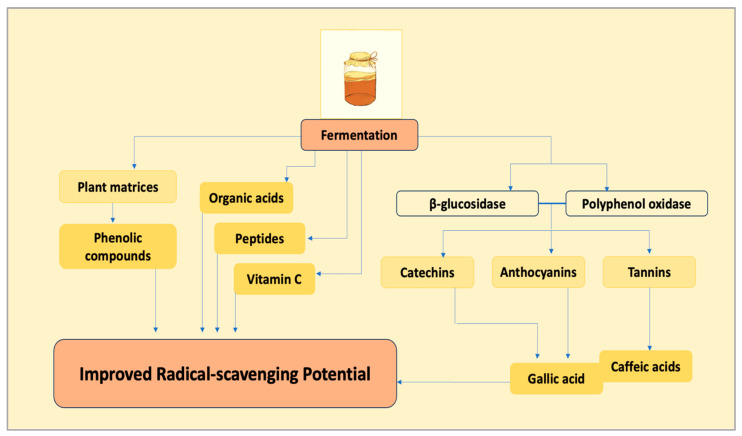
Key metabolites produced during fermentation are responsible for radical-scavenging potential of kombucha.

Key microbial enzymes, such as *β*-glucosidase and polyphenol oxidase, catalyze the depolymerization and deglycosylation of catechins, anthocyanins, and tannins, facilitating the formation of compounds such as gallic and caffeic acids, which exhibit strong radical-scavenging properties [14,61]. Fermentations employing fruit substrates, hibiscus flowers, or beetroot often show enhanced antioxidant activity; nevertheless, these effects are not universal and depend on substrate type, fermentation conditions, and analytical methodology. These enhancements are consistent with the production of key metabolites of kombucha as shown in Figure 3.

### 4.2. Anti-Inflammatory and Immunomodulatory Effects

The anti-inflammatory activity of kombucha is closely interconnected with its antioxidant capacity and involves the modulation of specific signalling pathways. Phenolic metabolites, such as gallic, ellagic, and chlorogenic acids, can inhibit NF-κB and COX-2 activation, resulting in decreased expression of pro-inflammatory cytokines, including TNF-α and IL-6, as demonstrated in in vitro cellular models [20,62]. Kombucha produced from herbal substrates, particularly *Hibiscus sabdariffa*, ginger, and mint, demonstrates strong suppression of inflammatory mediators, likely enhanced by fermentation-driven deglycosylation processes that increase the bioavailability of active compounds [57,63].

Emerging evidence highlights the immunomodulatory potential of these compounds. For instance, kombucha derived from pineapple by-products has been shown to reduce inflammatory markers and modulate immune cell activity in ex vivo models, although these findings should be interpreted cautiously, as ex vivo systems provide only a limited representation of in vivo immune responses [64].

### 4.3. Metabolic and Hypoglycemic Effects

Kombucha has been increasingly linked to beneficial effects on glucose and lipid metabolism, largely attributed to fermentation-derived organic acids, such as acetic and glucuronic acids, which influence insulin sensitivity, hepatic lipid turnover, and glycemic response; however, this relationship is primarily supported by studies on vinegar, and its direct relevance to kombucha remains to be fully established [57,62]. Kombucha produced from *Rhizophora mucronata* leaves demonstrated significant *α*-glucosidase inhibition, indicating potential hypoglycemic activity and supporting the role of phenolic–enzyme interactions in postprandial glucose regulation [40].

Similarly, kombucha derived from fruit byproducts, such as grape pomace and coffee silverskin, has shown enhanced lipid-lowering potential and improved cholesterol efflux, effects linked to elevated catechin and chlorogenic acid concentrations [23]. These findings suggest that metabolic outcomes can be modulated through substrate selection, with specific matrices supporting the targeted enrichment of bioactive acids and phenolics.

### 4.4. Probiotic Potential and Modulation of Gut Microbiota

Beyond its chemical constituents, kombucha may exert probiotic and prebiotic effects through its microbial population and extracellular polysaccharides (EPS). It should be noted that the probiotic status of kombucha remains debated, as most studies lack strain-specific identification and clinical validation required to meet established probiotic definitions. Although SCOBY-associated microorganisms differ from classical probiotic genera, several *Komagataeibacter* species and selected yeasts exhibit some resilience under simulated gastrointestinal conditions, although evidence remains limited and largely based on in vitro models, and their viability and functional activity in vivo are not yet well established [19,20].

EPS and bacterial cellulose produced during fermentation can function as prebiotic substrates that could support the proliferation of beneficial *Lactobacillus* and *Bifidobacterium* strains, although direct evidence in kombucha systems remains limited [63]. Fermentation of cereals and other plant-based matrices further enhances short-chain fatty acid production, particularly acetate, propionate, and butyrate, which contribute to improved intestinal barrier integrity and immunomodulatory activity [16,17]. These combined effects suggest that kombucha may play a complementary role in modulating gut microbial ecology and metabolic signalling.

### 4.5. Integrative Overview: Linking Biotransformation to Functionality

**Figure 4:**
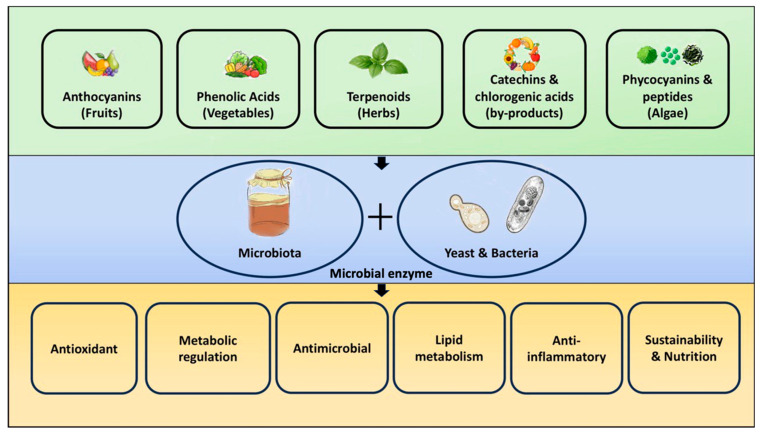
Functional spectrum of kombucha.

The functional spectrum of kombucha can be interpreted through the lens of substrate-driven microbial biotransformation, where SCOBY-derived enzymes convert plant precursors, such as sugars, phenolics, pigments, and peptides, into more bioavailable and biologically active metabolites (Figure 4). As different substrates possess distinct biochemical profiles, their transformation during fermentation yields characteristic and functional outcomes.

Together, these patterns demonstrate that kombucha fermentation can be strategically engineered for targeted functionality by aligning the substrate composition with the desired bioactive outcomes, a concept increasingly referred to as bioactive tailoring in functional beverage design. Emerging evidence indicates that fermentation conditions, such as temperature, duration, and microbial activity, interact closely with substrate characteristics to determine antioxidant, anti-inflammatory, metabolic, probiotic, and cosmetic potential. Table 2 provides an overview of fermentation parameters for kombucha beverages from a variety of alternative substrates along with their main characteristics and analytical methods used to established the main bioactive effects. Table 2 summarizes fermentation parameters reported in the literature; nevertheless, these conditions are highly heterogeneous and not standardized, which limits direct comparison across studies. Some reported conditions, such as elevated fermentation temperatures (e.g., 37 °C), deviate from typical SCOBY fermentation ranges and should be interpreted with caution.

## 5. Technological and Formulation Aspects of Kombucha Fermentation

The technological design of kombucha fermentation is critical for determining the biochemical quality, microbial stability, and functional potential of the final beverage. Compared with traditional *Camellia sinensis* infusions, alternative substrates introduce greater compositional and microbiological diversity, which markedly influences fermentation kinetics, enzyme activity, and the preservation of bioactive compounds. Therefore, optimizing the substrate formulation and process parameters is essential for maintaining both product stability and bioefficacy [24,79].

### 5.1. Influence of Substrate Composition on Fermentation Kinetics

The biochemical composition of the substrate, particularly its sugar profile, phenolic content, nitrogen availability, and buffering capacity, directly shapes microbial growth, pH evolution, and metabolite formation. Plant matrices rich in organic acids and phenolics, such as hibiscus, pomegranate, and jujube, tend to accelerate early fermentation by stimulating yeast glycolytic activity and enhancing acetic acid bacterial oxidation pathways [80,81]. These substrates often exhibit faster sugar depletion, more rapid pH decline, and increased production of organic acids. In contrast, substrates with low levels of fermentable sugars may require supplementation (e.g., sucrose addition) to ensure efficient microbial activity and stable fermentation dynamics.

Conversely, matrices containing higher protein, fiber, or polysaccharide content, such as oats, soy, or spirulina, frequently display slower acidification and limited ethanol turnover. In such systems, pretreatment steps (such as enzymatic hydrolysis or mild thermal processing) are often required to increase the availability of fermentable carbohydrates [24].

Sustainable substrates, such as coffee husks (cascara), grape pomace, and fruit peels, offer similar advantages. These byproduct-based fermentations often show high phenolic stability, strong antioxidant potential, and favorable kinetics when appropriately formulated [82,83].

### 5.2. Control of Process Parameters and Their Impact on Metabolite Profile

Process parameters, including temperature, fermentation duration, aeration, and inoculum ratio, play a decisive role in shaping the metabolic landscape of kombucha (Table 3). Optimal biofunctional quality is generally achieved at 25–30 °C with a fermentation period of 7–14 days, although the precise duration depends on substrate complexity and initial sucrose concentration [54,79,84]. In addition to primary fermentation, secondary fermentation (conditioning) is often applied to enhance carbonation, flavor development, and metabolite maturation, particularly in formulations based on alternative substrates. Higher temperatures accelerate acidification and SCOBY biomass formation but may promote the degradation of thermolabile phenolics, such as catechins and vitamin C [79]. In contrast, lower temperatures preserve phenolic integrity and produce beverages with smoother sensory profiles and extended shelf lives [39].

pH evolution is another key determinant of fermentation safety and its stability. Achieving a pH below 4.0 during the initial fermentation phase is crucial for inhibiting spoilage organisms and maintaining the microbial balance [85]. Oxygen availability further regulates the metabolic interplay within SCOBY: yeasts predominantly generate ethanol in oxygen-limited zones, whereas acetic acid bacteria oxidize ethanol to acetic, gluconic, and glucuronic acids in oxygen-rich layers. These organic acids are central to the detoxifying, antimicrobial, and hepatoprotective activities of kombucha [79] (Table 3). In controlled fermentation systems, such as bioreactors, oxygen availability can be actively regulated through aeration strategies, enabling more consistent microbial activity and metabolite production compared to traditional static fermentation.

**Table 3:** Key technological parameters affecting kombucha fermentation outcomes.

Parameters	Typical Range	Major Effects	Representative Studies
Temperature	25–30 °C	↑ Acids and vitamins; >30 °C → ↑ acetic acid and ethanol, may spoil; <20 °C → slower fermentation	[79,84,86]
Fermentation time	7–14 days	↑ Antioxidants and organic acids; too long → harmful acids ↑, nutrients ↓	[79,84]
Initial sucrose	3.17 g/100 mL	Vital nutritional source for the SCOBY to support its metabolic functions.	[87]
pH < 4.0	2.5–4.2	pH level ↓ when total organic acids content ↑, contributed to microbial stability	[59,85]
Oxygen supply	---	Yeast → ethanol; bacteria → acetic, gluconic, glucuronic acids → detox and liver protection.	[79]

Abbreviation: SCOBY (Symbiotic Culture of Bacteria and Yeast); ↑—increase; ↓—decrease.

### 5.3. Formulation Strategies to Preserve Bioactivity

Post-fermentation treatments strongly influence the stability of bioactive compounds in kombucha. Although thermal processing is necessary for microbial safety, it can significantly reduce the concentrations of heat-sensitive metabolites, such as phenolics and vitamins. Therefore, mild stabilization approaches, including low-temperature pasteurization (<60 °C) or microfiltration, are preferred to retain functional integrity while ensuring safety [39,88].

Secondary fermentation or controlled maturation can enhance carbonation and promote phenolic polymerization, resulting in improved antioxidant persistence, particularly in fruit-based formulations [81]. Innovative formulation strategies, such as the encapsulation of phenolics or probiotic cells within alginate or bacterial cellulose matrices generated from SCOBY biomass, further improve compound stability and enable targeted release in functional beverages [83,89].

Packaging technology also plays a crucial role. Containers with high light and oxygen barriers, such as amber glass or UV-protected PET, help mitigate oxidative degradation and maintain antioxidant capacity throughout storage.

### 5.4. Functional Product Development and Consumer Acceptability

Technological optimization must be balanced with sensory attributes and consumer expectations of the final product. Alternative substrates exert a strong influence on the sensory profile of kombucha: hibiscus and beetroot contribute vivid color and floral acidity, whereas algae- or moringa-based formulations may require natural flavor correction to enhance palatability [24,80]. Substituting refined sucrose with natural sweeteners, such as honey or fruit concentrates, can lower the glycemic load while improving microbial adaptability and fermentation performance [88,90].

Hybrid formulations that combine kombucha with fruit juices, herbal infusions, or plant milk alternatives are increasingly popular and align with consumer demand for “clean label,” low-sugar, and probiotic beverages [82,87]. The principal technological challenge is designing formulations that maintain microbial vitality, preserve functional metabolites, and deliver consistent sensory quality throughout the shelf life.

By fine-tuning the substrate composition, controlling the process variables, and applying gentle stabilization measures, manufacturers can achieve reproducible biofunctional profiles while meeting sensory and market expectations. The integration of microbial ecology, process engineering, and functional design positions kombucha as a versatile framework for developing next-generation fermented beverages tailored to specific health-promoting properties [24,79].

## 6. Challenges, Limitations, and Future Perspectives

Although research and commercial interest in kombucha produced from alternative substrates is rapidly expanding, several scientific, technological, and regulatory challenges continue to limit its standardization and broader application. Key limitations are methodological inconsistencies, insufficient mechanistic understanding, uncertainties regarding bioavailability, and the difficulty of achieving scalable, safe, and sustainable production systems [56,85,91]. Addressing these issues will require coordinated interdisciplinary efforts integrating food technology, microbiology, systems biology, and bioprocess engineering.

### 6.1. Lack of Process Standardization and Reproducibility

One of the most significant barriers to advancing kombucha science is the lack of standardized fermentation protocols. Studies vary widely in fermentation duration (5–21 days), temperature (20–35 °C), substrate concentrations, aeration conditions, and inoculum ratios, often without detailed reporting of pH evolution or oxygen exposure [85,91]. These inconsistencies lead to substantial variations in sugar depletion, acid production, and microbial succession, making cross-study comparisons and meta-analyses difficult.

Another challenge is the undefined and heterogeneous nature of the SCOBY consortium. Microbial composition varies depending on geographical origin, substrate type, and propagation history, resulting in unique bacterial–yeast interactions in each culture [92]. Although high-throughput sequencing has improved the taxonomic characterization of SCOBY communities [85], the functional contributions of individual strains, particularly their impact on flavor development, cellulose biosynthesis, and metabolite formation, remain poorly understood.

Improving reproducibility will require the development of harmonized reporting standards, including pH trajectories, redox potential, oxygen diffusion, and full characterization of the inoculum microbiota. Establishing reference SCOBY strains or defined microbial starter cultures, analogous to those used in wine and dairy fermentation, would provide a valuable benchmark for consistent inter-laboratory comparisons [56].

### 6.2. Analytical and Mechanistic Limitations

Despite advances in chromatography, metabolomics, and spectroscopic profiling, the mechanistic understanding of kombucha fermentation remains limited. Most studies rely on global measurements, such as total phenolics or antioxidant activity (DPPH, ABTS), without linking specific molecular transformations to microbial metabolism [28,93]. Only a few investigations have employed multi-omics approaches, integrating metabolomics, metatranscriptomics, or proteomics, to track the enzyme-mediated conversion of polyphenols into new bioactive metabolites [55].

Furthermore, quantitative structure–activity relationships (QSAR) and computational pathway models are underdeveloped in this field. Establishing mechanistic links between microbial transformations and biological endpoints, such as anti-inflammatory or hypoglycemic activity, would greatly advance functional interpretation [40,64]. Emerging molecular docking and in silico metabolite prediction tools hold promise for elucidating these connections [38,94].

Future studies should adopt systems-level “bioprocess-omics” platforms that correlate microbial gene expression, enzymatic kinetics, and metabolite fluxes under controlled environmental conditions to help clarify how substrate composition and process parameters jointly determine the formation of the target metabolites.

### 6.3. Bioavailability and In Vivo Validation

A key limitation is the translational gap between in vitro bioactivity and in vivo or clinical effectiveness. Although kombucha contains diverse polyphenols, organic acids, and peptides, few studies have investigated their absorption, metabolism, or systemic effects in animal or human models [64,90]. Phenolic compounds undergo extensive transformation during digestion, and the resulting metabolites may differ markedly in bioactivity from their parent structures. Mechanisms of bioactive properties of kombucha, especially from a variety of fermentation substrates, remain largely unknown as well. Considering the presence of polyphenols in kombucha products and the epigenetic activity reported for polyphenols [95,96,97], including a CRISPR-Cas9-based mechanistic [98] it may be hypothesized that kombucha would exert effects on transcriptional regulation of genes. This knowledge would be vital for incorporating kombucha in precision approaches to health maintenance and disease prevention. Similarly, the survival, colonization potential, and functional effects of SCOBY-derived microorganisms remain poorly characterized. Acetic and lactic acid bacteria present in some kombucha strains show variable tolerance to gastric acidity and bile salts [99,100].

Future validation efforts should involve standardized gastrointestinal simulations and controlled human trials that measure functional biomarkers, such as oxidative stress, inflammatory markers, and gut microbiota modulation [101]. Integrating pharmacokinetic modeling with bioassay-guided metabolomics could help determine the bioavailability, metabolic fate, and effective dosage of kombucha products.

### 6.4. Technological Scalability and Formulation Challenges

Scaling up kombucha production while retaining its bioactivity and microbiological safety presents significant engineering challenges. Industrial systems must ensure stable oxygenation, temperature control, and contamination prevention. In addition, the management of cellulose-based biofilms (SCOBY structure) represents a critical challenge, as it affects mass transfer, process stability, and cleaning efficiency. Alternative substrates derived from agro-industrial residues or algae often display variable sugar profiles and buffering capacities, resulting in inconsistent fermentation kinetics [49,92].

Post-fermentation stabilization complicates production. Pasteurization can degrade thermolabile phenolics and vitamins, whereas unpasteurized kombucha may contain spoilage organisms and pathogens [56]. Mild thermal stabilization (<60 °C), microfiltration, and encapsulation of probiotics within bacterial cellulose matrices offer promising intermediate solutions [89].

Looking ahead, precision fermentation in bioreactors featuring immobilized SCOBY systems, controlled aeration, and digital monitoring of metabolite fluxes may enable continuous or semi-continuous production of standardized kombucha with defined functional profiles [94,102].

### 6.5. Sustainability and Valorization Opportunities

Kombucha fermentation strongly aligns with circular economy principles, offering sustainable pathways for food and agricultural byproducts. Substrates such as coffee husks, fruit pomace, and cereal bran provide abundant carbon sources while reducing waste streams [49,101]. Nevertheless, compositional heterogeneity and potential contaminants, such as pesticides or mycotoxins, require pretreatment and robust safety validation [29,83]. Sustainability evaluations should incorporate life-cycle assessment (LCA) and techno-economic modeling to determine environmental impact and feasibility [48]. SCOBY-derived bacterial cellulose is a valuable renewable biomaterial for textiles, wound dressings, and biodegradable packaging [103,104]. Therefore, integrating kombucha fermentation into broader bioprocessing systems could extend its utility well beyond beverage production.

### 6.6. Future Perspectives

The future of kombucha research and development is likely to be shaped by precision fermentation, synthetic microbiology, and digital bioprocessing. Engineering *Komagataeibacter* strains for improved cellulose synthesis, enhanced tolerance, or tailored phenolic metabolism may enable targeted functional beverage design [94,102] (Table 4). Real-time monitoring of fermentation through sensor technologies, metabolite profiling, and AI-driven predictive control could permit the dynamic optimization of microbial metabolism to achieve customized bioactive profiles [32,93].

Advances in encapsulation, hybrid fermentation, and plant-based formulations may drive the development of personalized, nutrient-dense functional beverages [28,58]. Beyond food applications, SCOBY-derived cellulose and secondary metabolites are emerging as promising inputs for biomedical devices, packaging materials, and biosensors [83,103].

In summary, although substantial progress has been made in understanding the microbial ecology and functional potential of kombucha, key challenges remain in methodological standardization, mechanistic elucidation, bioavailability assessment, scalable production, and sustainability integration. Overcoming these barriers will require collaborative multidisciplinary approaches that combine analytical chemistry, microbial systems biology, genomics, process engineering and computational modeling. Such convergence will be essential to unlocking the full potential of kombucha as both a functional beverage and a sustainable biotechnological platform.

## 7. Methodology

### 7.1. Literature Search Strategy

A semi-systematic literature search was conducted to identify studies investigating kombucha beverages produced using substrates other than *Camellia sinensis*. This approach ensures transparent and reproducible data collection while accommodating substantial methodological heterogeneity in substrate selection, fermentation protocols, and analytical techniques.

The search covered publications from January 2020 to September 2025 and focused on recent studies evaluating alternative substrates and their functional outcomes. Earlier publications were additionally included where necessary to provide fundamental background on kombucha microbiology, fermentation mechanisms, and biochemical transformations. The search was performed using major bibliographic databases, including PubMed, Scopus and Web of Science. In addition, relevant articles were identified through searches of publisher platforms, including ScienceDirect (Elsevier), as well as manual screening of journals published by MDPI. The following keyword combinations were used:

“kombucha” AND (“alternative substrate” OR “non-tea” OR “fruit” OR “herbal” OR “juice” OR “vegetable” OR “by-product”) AND (“bioactive” OR “antioxidant” OR “polyphenol” OR “functional beverage” OR “fermentation”).

Additional studies were identified through reference chaining and manual screening of the literature, as summarized in Supplementary Material S1. The search results were imported into the Mendeley Reference Manager for organization and duplicate removal. Two independent reviewers screened the titles, abstracts, and full texts according to the predefined eligibility criteria (Section 2.2). Because this review employed a narrative/semi-systematic format, no PRISMA flowchart was applied due to the heterogeneity of study designs and substrates.

### 7.2. Inclusion and Exclusion Criteria

Studies were included if they met the following criteria:

- Peer-reviewed articles published in English;
- Investigated kombucha fermented using substrates other than traditional *Camellia sinensis* tea (e.g., fruit, herbal, vegetable, microalgal, cereal, or food-industry by-products);
- Reported chemical, microbiological, technological, or functional outcomes relevant to substrate-specific modulation of bioactivity;
- Provided sufficient methodological or analytical details to enable qualitative comparison.

Studies were excluded if they

- Did not report fermentation-related or bioactive outcomes;
- Focused solely on sensory evaluation or consumer perception;
- Were non-peer-reviewed works (e.g., theses, conference abstracts, patents);
- Were not available in English.

Controlled human trials were not included in the main synthesis because of their different methodological scopes; instead, they are summarized separately in Supplementary Material S2 to provide clinical context.

### 7.3. Data Extraction and Synthesis

For each included study, information was extracted as follows:

- Substrate type and origin;
- Fermentation conditions (duration, temperature, inoculum characteristics);
- Analytical methods used to quantify phenolics, pigments, organic acids, antioxidant activity, and other metabolites;
- Bioactive properties (antioxidant, anti-inflammatory, metabolic, probiotic, or other functional effects).

The extracted data were qualitatively synthesized and organized into a comparative overview presented in Table 1, which summarizes the substrate categories and associated bioactive outcomes. Additional mechanistic and chemical details that exceed the scope of the main text are presented in Supplementary Material S2.

Given the high heterogeneity in fermentation conditions and analytical endpoints, no quantitative meta-analysis was conducted. Instead, a structured qualitative synthesis was conducted in accordance with the best practices for integrative biochemical and technological reviews.

### 7.4. Quality Assessment

An internal methodological checklist was used to assess the transparency and reproducibility of the included studies. The checklist evaluated the following:

- Clarity and completeness of fermentation parameters;
- Suitability and reporting of analytical methods;
- Statistical transparency (replicates, variance measures, controls).

Minimum methodological criteria for inclusion required that studies clearly described the fermentation process, specified at least one validated analytical method for bioactive or functional assessment, and provided sufficient methodological detail to allow qualitative comparison across studies. Studies lacking a clear description of fermentation conditions or analytical methodology were excluded from the synthesis.

Two reviewers independently assessed each study, and discrepancies were resolved through discussions. Only studies meeting these minimum methodological requirements were included in the final analysis.

### 7.5. Methodological Notes

Although this review is primarily narrative, it follows a structured semi-systematic (narrative) approach that combines elements of systematic literature searching with qualitative synthesis. The literature search, screening, and data extraction procedures were conducted using a defined and reproducible framework, including selected databases, keyword combinations, and inclusion/exclusion criteria. However, due to the substantial heterogeneity in substrate types, fermentation conditions, and analytical endpoints across studies, a fully systematic review or meta-analysis was not feasible. This hybrid approach enables meaningful comparisons across studies employing diverse substrate categories and analytical methodologies. The resulting synthesis integrates biochemical, microbiological, and technological evidence to illustrate how alternative substrates modulate the bioactive properties of kombucha.

Analytical techniques and bioactivity assays reported in the included studies were not evaluated as a separate methodological endpoint but were considered within a qualitative comparative framework. Differences in analytical approaches (e.g., spectrophotometric assays versus chromatographic techniques) were taken into account when interpreting variability in reported bioactive outcomes and their comparability across studies.

## 8. Conclusions

The expansion of kombucha fermentation beyond traditional tea matrices reflects the broader evolution of functional beverage biotechnologies. The evidence synthesized in this review demonstrates that alternative substrates, from fruits, vegetables, herbs, and algae to food industry by-products, profoundly influence microbial ecology, biochemical pathways, and the formation of bioactive metabolites. Through coordinated microbial and enzymatic biotransformations, these substrates yield phenolic acids, organic acids, peptides, and other metabolites associated with enhanced antioxidant, anti-inflammatory, metabolic, and probiotic activities.

The interplay between substrate composition and fermentation ultimately determines the chemical complexity and functional efficacy of the final product. In addition to broadening nutritional and sensory diversity, the use of alternative raw materials provides opportunities for the sustainable valorization of agricultural residues, aligning kombucha production with circular economy principles.

Despite this rapid progress, key challenges remain. Absence of standardized fermentation protocols limited mechanistic insight into substrate–microbe interactions, and insufficient mechanistic, in vivo and clinical validation of biological effects constrain the translation of laboratory findings into evidence-based dietary recommendations. Addressing these gaps will require the integration of advanced analytical methodologies, multi-omics profiling, precision fermentation strategies, and system-level bioprocess control.

Looking forward, the convergence of microbial ecology, biochemical understanding, and process engineering positions kombucha as more than just a traditional fermented beverage. It represents a versatile biotechnological platform capable of supporting next-generation functional foods designed for verified health benefits, sustainability, and consumer well-being.

## Figures and Tables

**Table 2:** Main bioactive effects and analytical methods used to establish these effects, along with fermentation parameters, for kombucha beverages obtained from a variety of alternative substrates.

Substrate Type	Main Bioactive Effect(s)	Analytical Methods and Bioactivity Assays	Fermentation Parameters	Reference
Black tea (control)	Baseline antioxidant and antimicrobial activity	DPPH, FRAP, ABTS, HPLC	28 °C, 10 days	[59]
*Hibiscus sabdariffa*, Ginger, Mint	High antioxidant and anti-inflammatory potential; rich in anthocyanins	DPPH, FRAP, LC–MS	30 °C, 7–10 days	[19,65]
Soy milk	High antioxidant activity	DPPH, ABTS, FRAP	37 °C, 3–4 days	[66]
Soy whey	High antioxidant activity	DPPH, ABTS, FRAP	28 °C, 8 days	[67]
Coffee	High antioxidant activity High oxygen radical absorbance capacity	DPPH, ORAC	23 °C, 8 days	[68]
Green tea	Anti-*Candida* activity Anti-fungal activity. High antibacterial activity	Agar well diffusion	20–30 °C, 14 days	[69]
Rooibos leaves	High antioxidant activity Improved cell viability	DPPH, FRAP, H_2_O_2_-induced oxidative stress	28 °C, 7–14 days	[68]
Yerba-maté	High antibacterial activity	Agar disc diffusion	25–30 °C, 12 days	[70]
Lemon balm	High total phenols and antioxidant activity, antibacterial activity	Agar well diffusion	28 ± 1 °C, 7 days	[71]
Purple basil (*Ocimum basilicum*)	High inhibition against DPPH,	DPPH, ABTS, CUPRAC assay	24 ± 3 °C, 10 days	[72]
Peppermint	high catalase activity High antioxidant activity	Catalase activity, DPPH, FRAP	28 ± 1 °C, 7 days	[73]
Oak leaves	High oxygen radical absorbance capacity and ABTS radical scavenging activity High NO scavenging activity against peroxyl and peroxynitrite anions	ABTS, ORAC, NO scavenging assay	25 °C, 7 days	[74]
Oolong tea	Potent radical scavenging activities. H_2_O_2_-induced ROS production Increase in mRNA expression of antioxidant en- zymes	DPPH, ABTS, mRNA expression, H_2_O_2_-induced ROS production	25–30 °C, 14 days	[69]
Yarrow (*Achillea millefolium*)	High antioxidant Properties Anti proliferative activity High activity against RD cells.	DPPH, ABTS, MTT assay, Wound-healing assay, mRNA expression	25 °C, 7 days	[75]
Arabica green coffee	lower DPPH-scavenging activity High SOD activity	SOD activity assay, DPPH	7–28 days	[76]
Acerola	High antioxidant activity	DPPH	30 °C, 15 days	[77]
River redgum	High antioxidant activity, high anti-inflammatory potential	DPPH, TBARS, NO	25 °C, 7 days	[78]

Abbreviations: DPPH (2,2-diphenyl-1-picrylhydrazyl), FRAP (Ferric Reducing Antioxidant Power), ABTS (2,2’-azino-bis (3-ethylbenzothiazoline-6-sulfonic acid)), LC–MS (Liquid chromatography–mass spectrometry), HPLC (High-performance liquid chromatography), ORAC (Oxygen Radical Absorbance Capacity), CUPRAC (Cupric Reducing Antioxidant Capacity), MTT (3-(4,5-dimethylthiazol-2-yl)-2,5-diphenyltetrazolium bromide), SOD (Superoxide dismutase), TBARS (Thiobarbituric Acid Reactive Substances), NO (Nitric oxide), ROS (Reactive Oxygen Species). Reported fermentation parameters reflect diverse experimental designs and are not standardized across studies. Typical SCOBY fermentation is generally conducted at 25–30 °C; deviations from this range may influence microbial dynamics and metabolite profiles.

**Table 4:** Future perspectives of kombucha research and development.

Research Domain	Current Challenges and Limitations	Future Directions and Technological Opportunities
Fermentation standardization	Non-uniform process parameters; undefined SCOBY composition [85,91]	Develop standardized fermentation protocols; define microbial starter cultures; report full physicochemical profiles
Analytical and mechanistic understanding	Limited integration of multi-omics; lack of QSAR or metabolic pathway mapping [55,93]	Apply combined metabolomics–transcriptomics; use AI-aided modeling to link metabolites with functions
Bioavailability and efficacy	In vitro assays dominate; minimal in vivo or clinical evidence [64,90]	Conduct animal and human trials; employ digestion models; integrate pharmacokinetics and metabolite tracing
Industrial scalability	Process heterogeneity; oxygen and pH control issues; safety–stability trade-off [49,89]	Design bioreactor-based precision fermentation; apply encapsulation and mild preservation technologies
Sustainability and circular economy	Unassessed environmental impact; variable waste-derived substrates [83,101]	Perform LCAs and techno-economic analyses; integrate kombucha by-products into biomaterial and packaging sectors
Next-generation innovation	Limited adoption of digital and synthetic biology tools [94,102]	Employ strain engineering, AI-driven optimization, and smart sensors for real-time fermentation control

Abbreviations: SCOBY (Symbiotic Culture of Bacteria and Yeast), LCA (Life Cycle Assessment), QSAR (Quantitative Structure–Activity Relationships).

## Data Availability

This article is a review and does not report original experimental data. All information analyzed is derived from previously published studies, which are cited in the reference list.

## Supplementary Materials

The following supporting information can be downloaded at https://www.mdpi.com/article/10.3390/molecules31071170/s1: Supplementary Material S1: Extended Review Data on Alternative Substrates Used in Kombucha Fermentation; Supplementary Material S2: Summary of Controlled Human Trials Evaluating Kombucha Consumption.

## Abbreviations

The following abbreviations are used in this manuscript:

SCOBY	Symbiotic Culture of Bacteria and yeast
EPS	Extracellular Polysaccharides
DPPH	2,2-diphenyl-1-picrylhydrazyl
FRAP	Ferric Reducing Antioxidant Power
ABTS	2,2’-azino-bis (3-ethylbenzothiazoline-6-sulfonic acid)
ORAC	Oxygen Radical Absorbance Capacity
LC–MS	Liquid chromatography–mass spectrometry
CUPRAC	Cupric Reducing Antioxidant Capacity
MTT	3-(4,5-dimethylthiazol-2-yl)-2,5-diphenyltetrazolium bromide
SOD	Superoxide dismutase
TBARS	Thiobarbituric Acid Reactive Substances
NO	Nitric oxide
ROS	Reactive Oxygen Species
TPC	Total phenolic content
T-SOD	Total superoxide dismutase
MDA	Malondialdehyde
PBMC	Peripheral blood mononuclear cells
ELISA	Enzyme-linked immunosorbent assay
LCA	life-cycle assessment
QSAR	quantitative structure–activity relationships
HPLC	High-performance liquid chromatography
