# Supplementary material for: Shaping the Bioactive Properties of Kombucha Drinks by Using Raw Materials Alternative to Tea
Source: Molecules. 2026 Apr 1;31(7):1170. doi: 10.3390/molecules31071170 (PMC13074635; doi:10.3390/molecules31071170)
Supplement: Supplementary file 1 [file molecules-31-01170-s001.zip › Chandran et al_Supplementary Material S1.pdf]

Supplementary Material S1. Extended Review Data on Alternative Substrates Used in Kombucha Fermentation

S1.1. Purpose of This Supplement

This document provides extended background, detailed tables, and additional explanations supporting the main findings of the review *"Shaping the bioactive properties of kombucha drinks by using raw materials alternative to tea"*. It includes:

- Expanded descriptions of substrate categories
- Additional mechanistic explanations
- Extended tables on metabolites and functional properties
- Clarifications that did not fit into the main text due to length limits

S1.2. Search and Selection Approach (Methodological Note)

A semi-systematic literature search was conducted to identify scientific studies examining kombucha produced from substrates other than *Camellia sinensis*. The search included:

- **Databases:** PubMed, Scopus, Web of Science, ScienceDirect
- **Years covered:** 2010–2025
- **Keywords:** "kombucha", "fermented beverage", "alternative substrate", "fruit kombucha", "herbal kombucha", "bioactive compounds", "SCOBY fermentation"
- **Inclusion criteria:**
    - studies involving fermentation using non-tea substrates
    - reporting chemical composition, microbial dynamics, metabolite formation, functional or antioxidant properties
    - peer-reviewed articles in English

This search strategy is intended to support a narrative/semi-systematic synthesis and not a full PRISMA-based systematic review.

S1.3. Extended Overview of Alternative Substrate Categories

Herbal and Botanical Substrates

- Include hibiscus, chamomile, rooibos, mint, mate, and medicinal plants.
- Typically increase flavonoid diversity, anthocyanins (hibiscus), and unique terpenoid profiles.
- Fermentation enhances organic acids and stability of phenolic constituents.

Fruit Substrates

- Strawberry, mulberry, grape, passion fruit, apple, and other fruits provide natural sugars and high phenolic content.
- Fermentation often increases total phenolic content (TPC), antioxidant capacity (DPPH, ABTS, FRAP).
- Anthocyanin-rich fruits show substantial biotransformation during fermentation (e.g., cyanidin → protocatechuic acid).

Vegetable Substrates

- Sweet potato leaves, carrot, beetroot, pumpkin, and leafy greens contribute distinct phenolic acids, minerals, and fibers.
- Fermentation promotes chlorogenic acid derivatives, carotenoids, and in some cases lactic acid bacteria growth.

Marine, Algae, and Seed Substrates

- Spirulina, chlorella, seaweeds, and seeds (e.g., hemp) diversify the nutritional composition through proteins, peptides, and pigments.
- Marine substrates may enhance total mineral content and introduce phycocyanins.

Food Industry By-Products

- Fruit pomace, coffee silverskin, brewery spent grain, and winery residues can be converted into kombucha-style beverages.
- Enable circular-economy applications and provide high anthocyanin, flavonoid, or chlorogenic-acid content.

S1.4. Extended Mechanistic Explanations

Microbial Dynamics

- Substrate chemistry modulates yeast:bacteria ratios in SCOBY.
- Polyphenol-rich substrates promote *Acetobacter* spp.; high-fiber matrices may favor *Lactobacillus* spp.
- Microbial cross-feeding accelerates phenolic biotransformation.

Metabolite Formation

- Organic acids: acetic, glucuronic, gluconic, lactic, succinic.
- Bioactive metabolites: chlorogenic acids, gallic acid, anthocyanin degradation products, peptides, carotenoids.

- Formation influenced by fermentation time, oxygen availability, sugar concentration.

S1.5. Safety and Quality Considerations

- pH should remain ≤3.5 to ensure microbiological stability.
- By-product substrates require screening for contaminants (heavy metals, pesticides).
- Ethanol content may vary depending on sugar load and fermentation time.

S1.6. Summary

Alternative substrates significantly influence the microbial ecology, metabolite formation, and bioactive profile of kombucha. This supplement provides expanded mechanistic insight and additional data that complement the main text.
