# Supplementary material for: Shaping the Bioactive Properties of Kombucha Drinks by Using Raw Materials Alternative to Tea
Source: Molecules. 2026 Apr 1;31(7):1170. doi: 10.3390/molecules31071170 (PMC13074635; doi:10.3390/molecules31071170)
Supplement: Supplementary file 1 [file molecules-31-01170-s001.zip › Chandran et al_Supplementary Material S2.pdf]

Supplementary Material S2. Summary of Controlled Human Trials Evaluating Kombucha Consumption

S2.1. Purpose

This supplement provides a concise synthesis of all controlled human trials assessing health outcomes related to kombucha consumption. It is included to offer clinical context and illustrate the current limitations of human evidence relative to the much broader preclinical literature on substrate-driven bioactivity.

S2.2. Overview Table of Controlled Human Trials (2020–2025)

Study	Design	Participants	Duration	Key Outcomes
Atkinson 2023	Acute, crossover	11 healthy adults	single-day	↓ GI and II after kombucha vs control
Mendelson 2023	Randomized, double-blind, crossover	12 adults with T2D	4+4 weeks	↓ fasting glucose vs baseline
Ecklu-Mensah 2024	Parallel RCT	24 healthy adults	4 weeks kombucha	modest microbiome shifts
Fraiz 2024 (Foods)	Parallel RCT	59 overweight adults	10 weeks	no major microbiota changes; ↓ GI symptoms
Fraiz 2024 (Nutrients)	Parallel RCT	same cohort	10 weeks	↓ IL-6 increase; altered salivary microbiota

S2.3. Key Cross-Study Observations

- Evidence is **limited**, with small sample sizes and short interventions.
- Effects are **modest** and highly dependent on product composition.
- Most studies use **tea-based kombucha**, not kombucha from alternative substrates.
- Human trials do **not yet confirm** many of the strong in vitro / in vivo functional effects observed in substrate-specific research.
- Safety reporting is generally incomplete but no major adverse events were described.

S2.4. Relevance to This Review

Although clinical trials do not investigate alternative substrates directly, they:

- provide context on realistic human responses,
- illustrate variability in kombucha effects,
- highlight gaps in translational research,
- underscore the need for standardized formulations.

This supplement therefore complements the review by distinguishing **established clinical effects** from **substrate-driven bioactive mechanisms**, which are still mainly studied in preclinical models.

S2.5. Summary

Human evidence for kombucha remains preliminary. Controlled trials show small but promising effects on glucose regulation, inflammation, and microbiota composition. Translating substrate-specific biochemical enhancements into demonstrated clinical benefits requires further research.
